# Solid papillary carcinoma of the breast

**DOI:** 10.4322/acr.2021.352

**Published:** 2022-01-13

**Authors:** Toyaja Jadhav, Shashi Shekhar Prasad, Bhupesh Guleria, Manvir Singh Tevatia, Prerna Guleria

**Affiliations:** 1 Command Hospital (Southern Command), Department of Pathology, Pune, Maharashtra, India; 2 Command Hospital (Southern Command), Department of Medical Oncology, Malignant Diseases Treatment Center, Pune, Maharashtra, India; 3 Command Hospital (Southern Command), Pune, Maharashtra, India

**Keywords:** Carcinoma, Papillary, Breast Neoplasms, Unilateral Breast Neoplasms, Carcinoma

## Abstract

Solid Papillary Carcinoma (SPC) of the breast is a rare tumor with an incidence of less than 1%, mainly affecting elderly females. It is morphologically characterized by well-defined nodules with low-grade nuclear features associated with fibrovascular cores and shows neuroendocrine differentiation. SPC can be in-situ or invasive but has a favorable prognosis. It is a morphological mimicker of some pre-malignant conditions leading to its frequent misdiagnosis. An appropriate immunohistochemical (IHC) panel workup helps in distinguishing this tumor from its various morphological mimics. In this report, we present one such case of SPC with a small focus of invasion, reviewing the literature.

## INTRODUCTION

Solid papillary carcinoma (SPC) is an uncommon malignancy of elderly females. It has an incidence of less than 1%, with the mean age of presentation being 70 years.^1^ It is said to originate from expanded ducts and mostly involves the central region of the breast. Morphologically, it is composed of well-circumscribed nodules with fibrovascular cores along with the presence of low-grade ductal cells. Their microscopic appearance may often be misinterpreted for other lesions such as florid ductal hyperplasia, lobular neoplasia, intracystic papillary carcinoma, and low nuclear grade ductal carcinoma in situ (DCIS).^2^ It usually follows an indolent behavior unless it is associated with invasion. The WHO classification (5th edition, 2019)^3^ considers carcinoma in situ for staging purposes without demonstrable or doubtful invasive foci.^3^ It has been documented that upfront metastasis is found in only 0.4% of cases and about 90% are localized lesions.^4^ On extensive literature search on Medline, PubMed, and Scopus,^4^ 296 cases have been reported to date among pre and post-menopausal females. We hereby present one such case, which was referred to our tertiary care center.

## CASE REPORT

**Figure 1:**
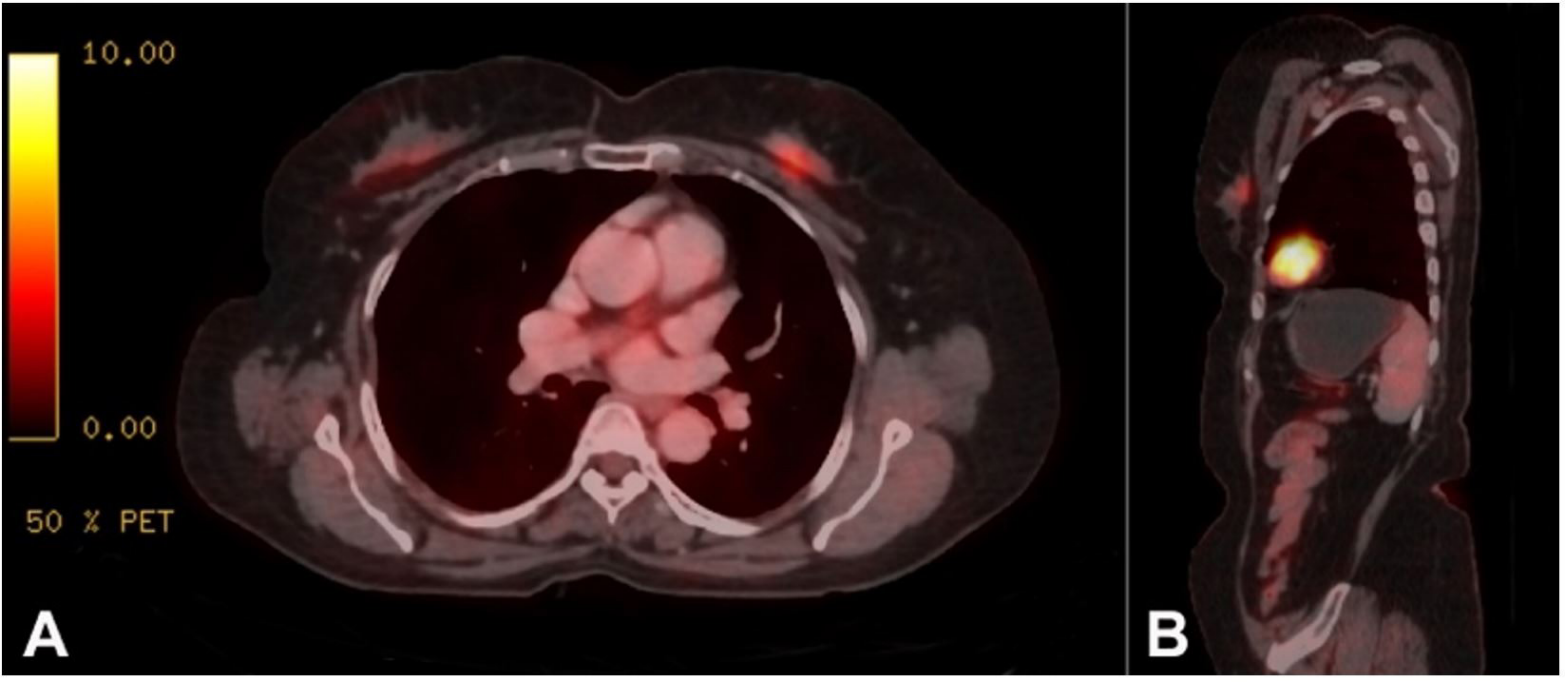
FDG-PET scan shows an ill-defined, hypermetabolic, heterogeneously enhancing FDG avid soft tissue nodular lesion involving upper inner quadrant of the left breast in axial view (Figure 1A) and parasagittal view (Figure 1B).

A 65-year-old female, with no family history of any malignancy, had presented with a palpable lump in the left breast (upper inner quadrant) associated with bloody nipple discharge of 3 months duration at a peripheral hospital. Sono-mammography revealed a solitary, solid, and incompressible hypoechoic nodule in the retroareolar region, measuring 17.5 x 9 mm, with heterogeneous echotexture and irregular margins, consistent with Breast Imaging Reporting and Data System (BIRADS).^3^ The patient underwent fine-needle aspiration cytology (FNAC) of the lesion, which was suspected of malignancy. Subsequent core biopsy of the lump was diagnosed as Invasive Lobular Carcinoma. FDG-PET scan revealed an ill-defined, hypermetabolic, heterogeneously enhancing soft tissue nodular lesion involving the upper inner quadrant of the left breast measuring 13 x 9 x 25 mm, with SUV max 4.6 (Figure 1).

No FDG avid lymph nodes were identified in bilateral axillae. She underwent modified radical mastectomy along with left axillary lymph node dissection, and the histopathology was reported as lobular carcinoma-in-situ.

**Figure 2:**
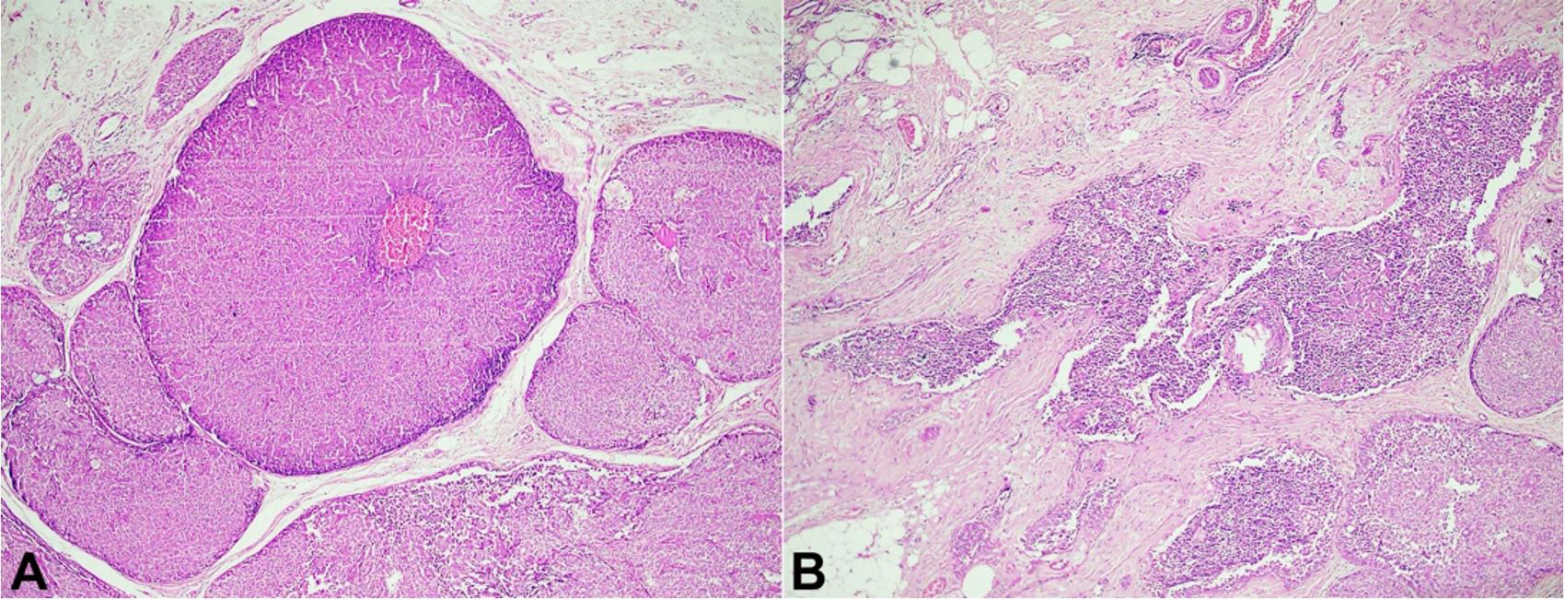
Photomicrograph of the tumor. **A** – reveal a tumor arranged in circumscribed large cellular nodules, closely apposed and expanded, separated by bands of fibrovascular stroma. The tumor cells are round to oval with moderate pleomorphism with a central vascular core (H&E, 400X); **B** – Shows foci of stromal invasion (H&E, 100X).

The patient was referred to our center for further management and review of the paraffin-embedded blocks of the resected specimen. The hematoxylin-eosin (H&E) stained sections revealed a tumor arranged in circumscribed large cellular nodules, closely apposed and expanded, separated by bands of the fibrovascular stroma. These nodules had foci of tumor arranged in papillae with fibrovascular cores. The tumor cells were predominantly round to ovoid, most of them showing moderate nuclear pleomorphism and granular eosinophilic cytoplasm with inconspicuous nucleoli. Occasional pseudo rosette formation was seen. However, no cellular palisading was noted. Areas of the extracellular matrix along with foci of stromal invasion were also present (Figure 2).

**Figure 3:**
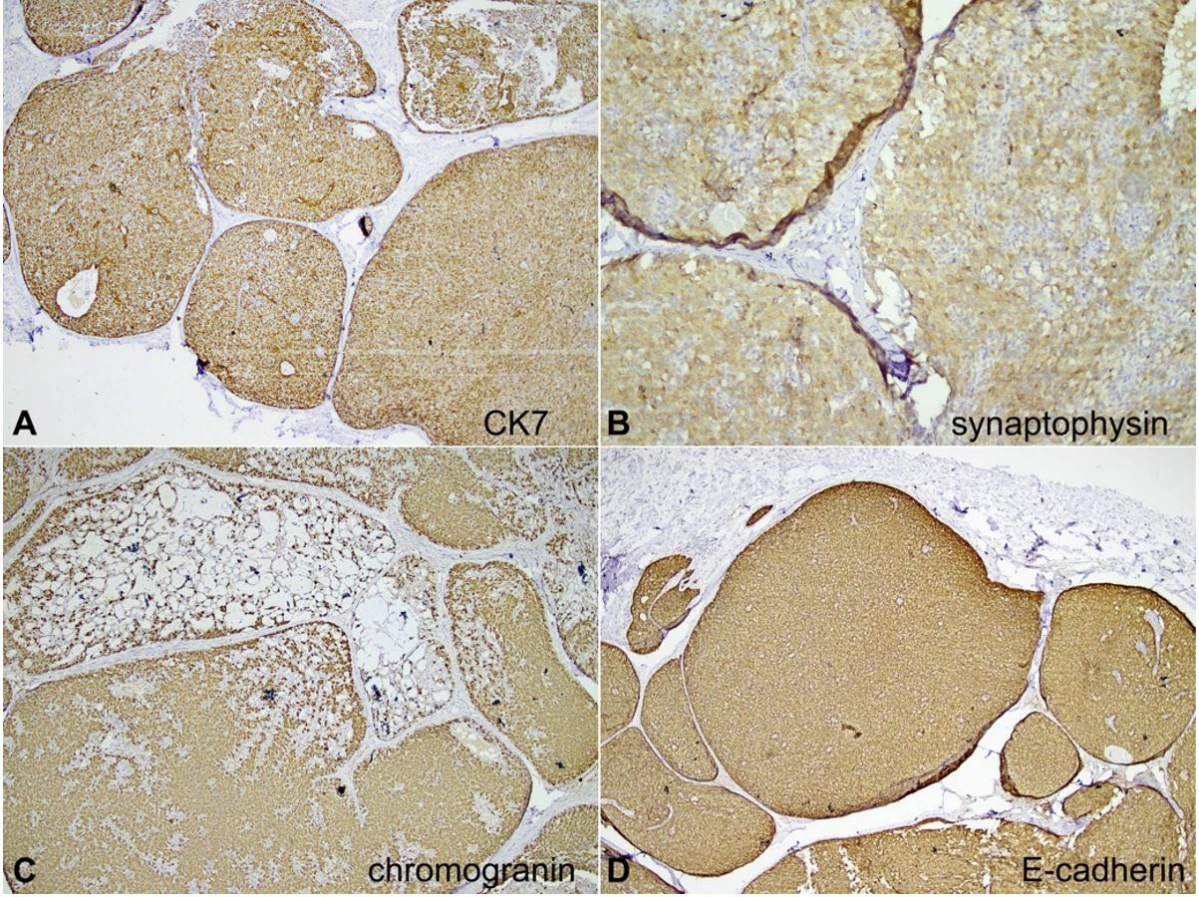
Photomicrographs of the tumor. Immunohistochemical panel. **A** – Strong immunoreactivity for cytokeratin (CK7, 40x); **B** – positivity to Synaptophysin (100x); **C** – positivity to chromogranin (40x); **D** – E-cadherin retained within the tumor cells (40x).

**Figure 4:**
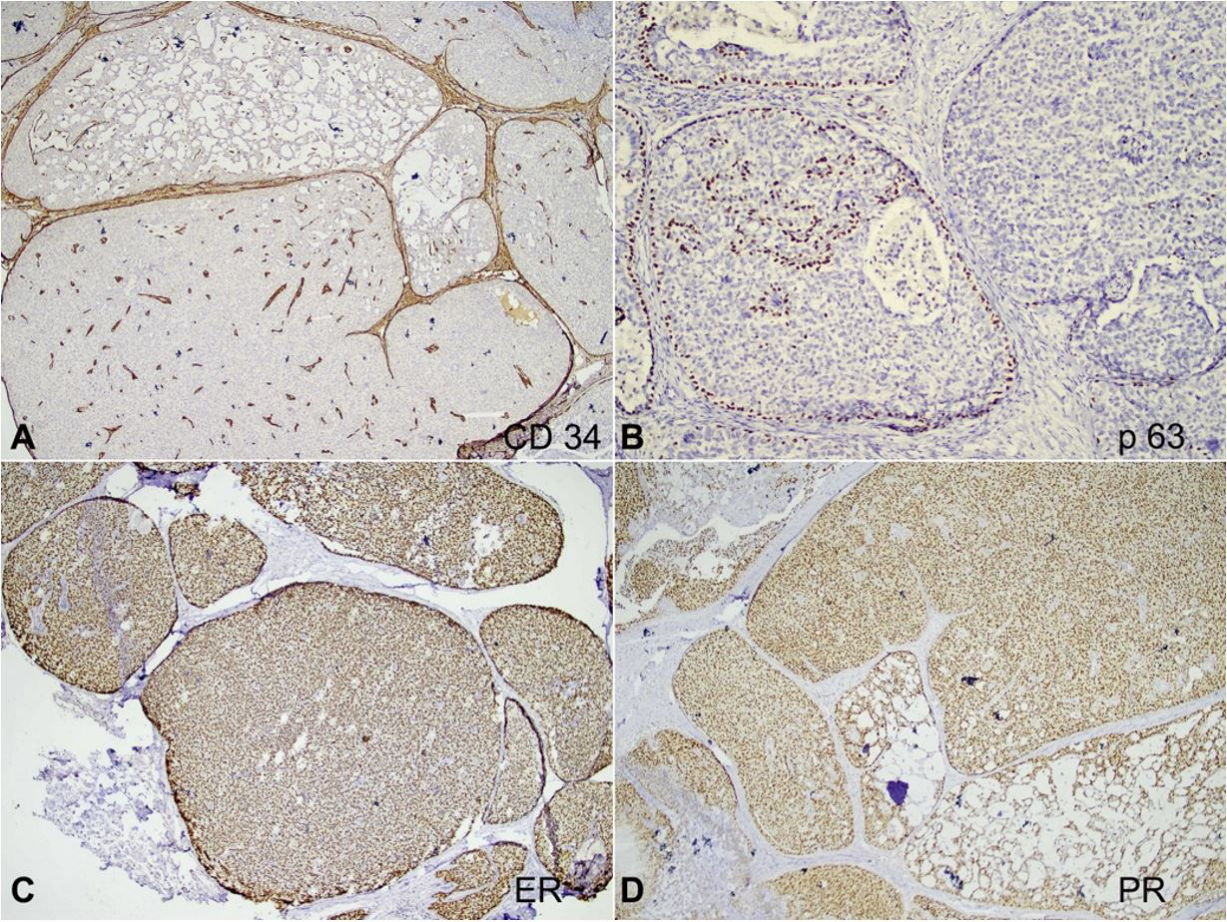
Photomicrographs of the tumor. Immunohistochemical panel. **A** – CD34 highlights the intermixed blood vessels within the tumor (40x); **B** – p63 shows focal loss of myoepithelial cells along the invasive foci (40x); **C** and **D** – Breast biomarker studies reveal immunopositivity for Estrogen Receptor (ER) (Allred score 8/8) and Progesterone Receptor (PR) (Allred score 8/8) respectively (40x).

**Figure 5:**
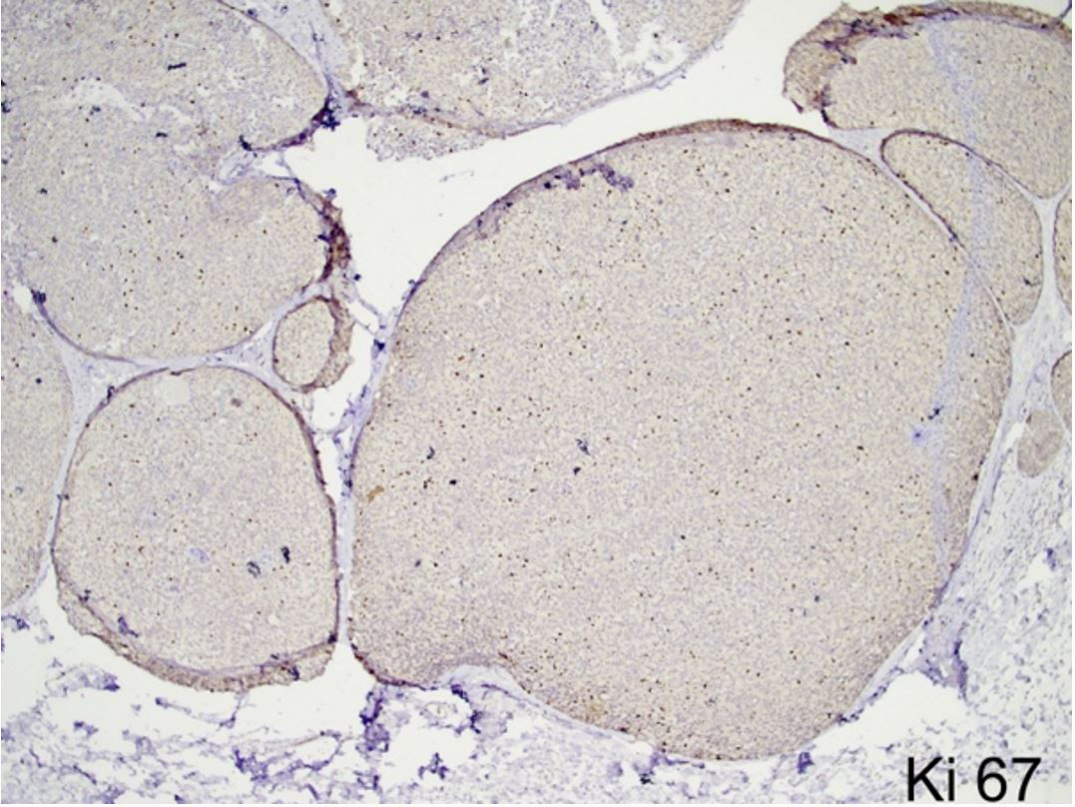
Photomicrographs of the tumor. Low to moderate Ki67 proliferation index of the tumor with 15-20% reactivity (40x).

On the immunohistochemical panel reactions, the tumor cells showed strong reactivity for cytokeratin (CK7), synaptophysin, and chromogranin. E-cadherin was retained within the tumor cells. CD34 highlighted the intermixed blood vessels while p63 showed focal loss of myoepithelial cells along with the invasive foci. Breast biomarker studies revealed immunopositivity for estrogen receptor (ER) (Allred score 8/8) and progesterone receptor (PR) (Allred score 8/8), and negativity for Her2 (ASCO/CAP guideline IHC score 0)^5^ (Figure 3 and 4). The Ki-67 was 15-20% (Figure 5). Hence, a final diagnosis of SPC of the breast (pT2N0) with foci of invasion into the surrounding stroma was rendered. Given the favorable histology and pT2N0 stage, she is currently on adjuvant endocrine therapy.

## DISCUSSION

In 1956, the term “Solid Papillary Carcinoma” was first proposed by Maluf and Koerner^6^ to describe a distinctive breast lesion, occurring especially among the elderly females and microscopically characterized by solid cellular proliferation of neoplastic cells supported by fibrovascular cores and forming circumscribed nodules.^7^ These neoplastic cells are monomorphous and present low-grade cytological features along with neuroendocrine differentiation. Most of these tumors show increased cellular proliferation, thereby masking the basic papillary properties, hence the name.

It is an uncommon breast lesion and constitutes approximately 1% of breast tumors.^4^ It is known to arise from the ducts and is considered a variant of ductal carcinoma in situ (DCIS) by some authors.^8^ These are well-defined lesions and can have mixed cystic and hemorrhagic components. The mean age of presentation is the 7th decade of life; however, it can also affect younger women. Our patient was 65 years old. About 95% of the tumors are unilateral, and approximately 50% arise in the retro-areolar or the subareolar regions; hence, most cases present as a centrally located breast mass with nipple discharge.^9^ Our patient also presented with an inner quadrant breast mass with bloody nipple discharge.

Mammography detection rates of this tumor range around 50%. Thus, many of the tumors may be mammographically occult.^10^ Ultrasonography detects around 50% of these tumors as BIRADS-4 or BIRADS-5 lesions. The ultrasonographic features include “frond-like” mass, either solid or complex cystic, within a dilated duct.^11^ The magnetic resonance imaging (MRI) is another radiological investigation tool with high sensitivity for detecting SPCs.^10^ A study has highlighted some characteristic features of SPC on MRI, including circumscribed lesion’s margins with heterogeneous signal intensity and rapid enhancement in the initial contrast phase and high apparent diffusion coefficient values with the absence of choline peak. These features may help to distinguish SPC from other invasive breast malignancies.^12^ The sono-mammography features of our case showed a hard and incompressible left breast mass with heterogeneous echotexture and irregular margins with spiculations, without any cystic changes or necrosis, consistent with BIRADS III lesion. Our patient was not submitted to an MRI study. FDG-PET scan, which is mainly done for the staging, restaging, and evaluation of treatment response in breast cancers, revealed a hypermetabolic soft tissue nodular lesion in the upper inner quadrant of the left breast with no other significant metabolic active disease elsewhere in the body. She was misdiagnosed as a case of ILC on a small biopsy.

Microscopically, SPCs are seen as multiple circumscribed nodules, each representing expanded ducts filled with neoplastic cells. In most cases, these nodules are composed of monomorphic cells with a low to intermediate nuclear grade. Sometimes the cells may appear spindled, and at other times may also appear plasmacytoid with granular eosinophilic cytoplasm and eccentrically-placed nuclei. The tumor nodules appear non-invasive due to their circumscription, and the demonstration of invasion becomes difficult at times.^2^^,^^3^ The papillary architecture is generally not apparent, but pseudo rosettes and nuclear palisading around stromal cores may be identified. Rarely, signet ring morphology may be seen.^2^^,^^13^

SPC characteristically shows intracellular mucinous differentiation, which, when present, clinches its diagnosis. Extracellular mucin production can also be seen; however, such foci need to be differentiated from invasive mucinous carcinoma.^13^ Mucinous carcinoma, Capella type B, characterized by large sheets of tumor cells with mucin production and neuroendocrine features, may resemble SPC to a large extent, especially when associated with less mucin production (≤50% mucinous component; a poor prognostic factor).^14^

There are two types of invasion patterns seen in SPC: SPC associated with conventional type of invasive carcinoma, where the invasive component may be composed of a pure invasive ductal carcinoma (IDC), or a mixed morphology composed of mucinous, neuroendocrine-like, IDC, or uncommonly, lobular and tubular subtypes. The invasive carcinoma component is usually low to intermediate-grade in these cases and often shows cytological features similar to the adjacent SPC. The second pattern predominantly shows SPC but with features of stromal invasion, most commonly associated with stromal desmoplasia. In such cases, SPC is often multinodular and shows multiple duct-like structures. SPC is considered invasive when the tumor nests show a characteristic jigsaw growth pattern with ragged and irregular margins.^13^ Immunohistochemistry plays an important role in the diagnosis of SPC. Loss of myoepithelial layer highlighted by the immunohistochemical loss of p63 is necessary to distinguish it from ductal carcinoma in-situ (DCIS), and may also confirm areas of doubtful invasion. Our case showed the absence of myoepithelial layer within the invasive foci highlighted by the loss of p63. Immuno-negativity of p63 was also seen in many tumor islands, thereby ruling out the solid/ papillary variant of DCIS.

The other helpful immunohistochemical feature for the definite diagnosis is the neuroendocrine differentiation (NED) reported in more than half of all SPC cases.^3^^,^^15^ Even though NED in other types of breast carcinomas has been regarded as a poor prognostic marker, the same is not true for SPC.^15^^,^^16^ The NED demonstrable in SPCs may therefore be considered more of a diagnostic rather than a prognostic marker.

SPC in-situ may be mistaken for other common breast neoplasms, such as papilloma with florid ductal hyperplasia. This lesion has well-formed fibrovascular cores, loss of monomorphism of the neoplastic cells and positivity for high molecular weight cytokeratins, which is negative in SPC.^3^ This distinction becomes difficult in the presence of overlapping features seen in either entities or when the sample is limited, such as in a core biopsy. The CK5/6 immunopositivity and the NED helps to distinguish between the two.^17^

Sometimes the histomorphological features of SPC in-situ or SPC with invasion, which is not so apparent, strongly mimic lobular carcinoma in situ (LCIS) as had happened with our case. The distinction between the two needs to be definitely made since LCIS is only a pre-malignant lesion involving different treatment protocols. Histomorphologically, LCIS is primarily a lobulocentric proliferation of small uniform cells, which fill and distend most of the acini in the involved lobule. It commonly involves the terminal duct lobular units (TDLUs). It is composed of small, uniform, round, and loosely cohesive cells, with or without intracytoplasmic mucin vacuoles, dense cytoplasm, and distinct cell membranes. Nuclei are small, monotonous, and eccentric, lack significant atypia or mitoses, and often have inconspicuous nucleoli. Their architectural appearance is characteristically called “marbles in a bag” appearance. Immunohistochemically, LCIS has accompanying myoepithelial cells in various patterns highlighted by markers such as p63, S-100 or smooth muscle myosin heavy chain (SMMHC) and the loss of E-cadherin.^18^ The solid papillary variant of invasive lobular carcinoma (ILC), which has been found to have a subclonal origin from the classical ILC, also may cause a diagnostic dilemma.^19^ Therefore, the subtle histomorphological features of solid lobules of monomorphic cell population with areas of papillary architecture, intra and extra-cellular mucin along with the immunohistochemical profile showing NED helps establish a diagnosis of SPC.

Table 1 compares features between florid ductal hyperplasia with papilloma, LCIS and SPC.

**Table 1:** Comparison between SPC and its common mimics

	SPC	FDHWP	LCIS
Age group commonly affected	Post-menopausal females (7th -8th decades)	Menopausal females (5th decades)	Pre-menopausal females (4th decade)
Type of neoplasm	Malignant	Benign	Premalignant lesion (Risk factor)
Papillary architecture	Present	Present	Absent
Stroma	Dense collagenous stroma	Dense collagenous stroma	Dense collagenous stroma
Epithelial cell proliferation pattern	Solid or fenestrated	Solid or fenestrated	Evenly spaced loosely cohesive cells: marbles in a bag appearance
Cell population	Monomorphous	Mild cellular pleomorphism	Monomorphous
Cellular streaming	Present	Present	Not seen
Pagetoid spread	Uncommon	Absent	Commonly present
Myoepithelial cells	Lost along the invasive fronds	Always present	Present
Nuclei	Irregular nuclei with granular chromatin	Irregular nuclei with granular chromatin	Small nuclei with evenly distributed chromatin and inconspicuous nucleoli.
Nuclear palisading	Almost always present	May or may not be seen	Absent
Nucleoli	Small	Small	Small
Fibrovascular cores	Present	Absent	Absent
Mitosis	Moderate to high	Low	Absent to low
Mucin	Intra and extracellular mucin present	Absent	Intracytoplasmic mucin vacuoles
CK 5/6 immunohistochemistry	Negative	Strongly Positive	CK5 positive
Neuroendocrine markers	Positive	Negative	Negative
p120 catenin	Negative	Negative	Strong cytoplasmic reactivity

FDHWP= Florid Ductal Hyperplasia with Papilloma; LCIS= Lobular Carcinoma In Situ; SPC= Solid Papillary Carcinoma (breast)

Other lesions that this entity may mimic include atypical ductal hyperplasia (ADH) and intracystic papillary carcinoma. Intracystic papillary carcinoma is defined as a solitary, centrally located malignant papillary proliferation involving a cystically dilated duct.^20^ While ADH does not show the presence of fibrovascular cores microscopically, like those seen in SPC; intracystic papillary carcinoma is characterized by the presence of papillary fronds lined by cuboidal cells that often reveal higher nuclear-grade on cytology.^21^

SPC may also, less commonly, mimic low-grade DCIS. However, it is to be noted that low-grade DICS, including neuroendocrine DCIS, fails to show a monotonous morphology of cells; and these cells lack the plasmacytoid or spindle cell appearance as seen in cells of SPC. Moreover, the presence of mucin, branching fibrovascular stroma, and ducts encompassed by fibrosis are also not the features of DCIS.^21^

Recently, there have also been reports of invasive lobular carcinomas (ILC) with a solid-papillary growth pattern mimicking SPC, known as Invasive lobular carcinoma with solid and encapsulated papillary carcinoma growth pattern.^22^ These cases typically showed focal merging of solid-papillary areas with classic invasive lobular carcinoma at the periphery, coupled with the presence of in situ lobular carcinoma and absent neuroendocrine differentiation, which supported a diagnosis of ILC over SPC. However, a separate study, which also reported a similar case, demonstrated that both the tumor’s solid-papillary and classic lobular components shared a common CDH1 mutation and a number of copy number alterations. In addition, the solid-papillary component had an additional 20q gain and 1p loss that have been reported to occur in the solid variant of invasive lobular carcinoma. It, therefore, concluded that both the solid-papillary and a classic lobular component of the tumor shared a common clonal ancestry. The diagnosis of invasive lobular carcinoma was confirmed by immunohistochemistry, which revealed negative E-cadherin, positive cytoplasmic P120, and deleted myoepithelium.^22^

With regards to breast biomarkers, SPCs show a luminal phenotype (estrogen and progesterone receptor positivity and Her2 negativity) with a relatively simple genome and a few copies of number alterations.^23^

Additional genetic features include loss of chromosome 16q and gain of chromosomes 1p and 16p. SPCs are also associated with a higher expression of genes attributing neuroendocrine differentiation, mainly *RET*, *ASCL1* and *DOC7*.^23^

Table 2 compares the various cases of SPC reported so far in the literature.

**Table 2:** Reported cases of SPC in the literature

Ref	# Of cases	Age years	Clinical features	Histology	Invasive component	NEd	ER/PR	Cytological features	Metastases
6	20	≥70	BL	SPC	-	+	PR+ve	Low grade	1 case: Lung
24	34	≥60	BL+ nd	niSPC	CC	+	ER+ve PR+ve	NA	NR
25	05	60	BL	BPTC	-	NA	NA	NA	NR
26	01	64	BL	BPTC	DCIS	NA	NA	Papillary Thyroid carcinoma	NR
27	21	66	BL	iSPC + niSPC	NEC	+	ER+ve PR+ve	NA	NR
8	20	≥60	Benign lesions +invasive Ca	SPC	NEC	+	ER+ve PR+ve	NA	NR
7	58	70	Benign lesions +invasive Ca	SPC Grade 1	NEC	+	ER+ve	NA	22 cases: lymph node 1 case: Distant metastasis
28	11	48-78	BL	niSPC grade 2-3	IDC	NA	NA	NA	7 cases: Lymph node
29	04	45-80	BL + thyroid nodule	BPTC	-	NA	variable	NA	1 case: Lymph node
30	01	66	BL	BPTC	IPC	NA	Triple Negative	Intraductal papilloma	NR
31	22	≥60	BL	iSPC + niSPC		+	ER/PR+ve	NA	NR
32	30	60-70	BL	iSPC + niSPC		+	ER+ve PR+ve	Low-grade l	NR
33	01	65	BL	BPTC	-	NA	NA	NA	NR
34	02	44, 55	BL	iSPC	-	NA	Not done	Papillary carcinoma	NR
35	32	67	BL + nd	niSPC	-	+	ER+ve PR+ve	NA	1 case: Distant metastasis
36	01	77	BL + nd	BPTC	DCIS	NA	Triple negative	Intraductal papillary lesion	NR
37	13	51-70	-	SPCRP	-	NA	Variable	NA	NR
38	13	58-79	BL	BPTC	-	NA	Triple Negative	NA	01 case: lymph node
12	04	66-79	BL + nd	SPC	-	+	Not done	NA	-
39	01	72	BL + nd	iSPC + necrosis	-	+	ER+ve PR+ve	NA	Lymph node
40	01	82	nd	iSPC + pagetoid extension	-	+	Not done	NA	lymph node
4	01	46	BL	iSPC		+	ER+ve PR-ve		Lung, liver and bone metastasis
This case	01	65	BL + nd	iSPC	-	+	ER+ve PR+ve	suspicious for malignancy	0

BL= breast lump; BPTC= Solid Papillary Carcinoma resembling Tall cell variant of Papillary Thyroid Carcinoma; CC= colloid carcinoma, IDC= invasive ductal carcinoma, IPC= invasive papillary carcinoma, iSPC= invasive solid papillary carcinoma; NEC= neuroendocrine carcinoma, NEd= Neuroendocrine differentiation, ND=Nipple discharge, niSPC= non-invasive SPC; NR= Not Reported; SPC= solid papillary carcinoma; SPCRP= Solid Papillary Carcinoma with Reverse Polarity; 0= No metastasis; ER= Estrogen Receptor; PR= Progesterone Receptor; NA= Not available; +: Present; -: Absent; #: Number.

The treatment protocols of SPC are still not well-established and vary from breast-conserving surgery to mastectomy depending upon the extent of the invasive component with or without adjuvant endocrine/chemotherapy. SPC in-situ is considered a variant of DCIS and is staged as pTis and treated on similar lines. The tumor size of SPC with invasion is determined by the invasive component only. The National Comprehensive Cancer Network (NCCN) guidelines mention the consideration of adjuvant endocrine therapy for smaller tumor size SPC (pT1-T3) without lymph node involvement or pN1mi. Adjuvant chemotherapy is definitely indicated in node-positive tumors.^24^ SPC is a tumor with a favorable prognosis with limited lymph node metastasis observed in only the ones with invasion.^3^

## CONCLUSION

Solid Papillary Carcinoma is seen in older women with a favorable prognosis. It has a morphological overlap with various benign and malignant lesions. It requires a thorough clinical, radiological and immunohistochemical workup to reach a definite diagnosis so that appropriate therapy can be administered for maximum patient benefit.
